# RECENT INNOVATIONS, CHALLENGES, AND OPPORTUNITIES FOR PEER PROGRAMS TO ADDRESS LONELINESS IN OLDER ADULTS

**DOI:** 10.1093/geroni/igae098.1066

**Published:** 2024-12-31

**Authors:** Ashwin Kotwal, Katrina Hough, Diane Meier, Soe Han Tha, Nandini Singh, Shannon Fuller, Janet Myers, Carla Perissinotto

**Affiliations:** University of California San Francisco Medical Center, San Francisco, California, United States; University of California San Francisco, San Francisco, California, United States; Icahn School of Medicine at Mount Sinai, New York, New York, United States; University of California San Francisco, San Francisco, California, United States; University of California San Francisco, San Francisco, California, United States; Johns Hopkins University, Baltimore, Maryland, United States; University of California San Francisco, San Francisco, California, United States; University of California San Francisco, San Francisco, California, United States

## Abstract

Peer interventions which pair individuals of similar age or life experience are rapidly expanding to address the growing public health challenge of loneliness and social isolation among diverse older adults. We examined COVID-19 pandemic-era innovations, ongoing challenges, and future opportunities for growth across six peer programs across California. We conducted 65 qualitative interviews (August 2023 - March 2024) with current and former older adult clients (n=24), peer specialists (n=12), program leaders (n=12), and policy and thought-leader stakeholders (n=18). We identified two major innovations. First, programs developed flexible, multi-modal support across in-person, virtual, phone, and group activities which extended reach to older adults experiencing functional limitations, technological barriers, or unique social needs. Second, programs elevated peer roles on behavioral health teams and developed new partnerships with city and community-based programs. Primary challenges include uncertainty on how to balance hybrid programming, the reduced availability of volunteers, and reduced state funding since the pandemic. Stakeholders identified recent Medicaid reimbursement for peer services as promising for financial sustainability, and the potential for peers to address loneliness among several high-risk groups (unhoused, subsidized housing, homebound, dementia, caregivers, immigrants, and LGBTQ). However, peers need clear training, delineation of roles from other team members (social work, community health workers), and efforts to prevent “peer drift” or deviating from core elements of the program with new responsibilities. In conclusion, innovations in peer programs point to growing potential to address loneliness and social isolation among high-risk older adults, but programs require support in navigating financial, training, and infrastructure needs.

